# Diagnostic accuracy of the waist-to-height ratio and other anthropometric indices for metabolically healthy obesity in the working population

**DOI:** 10.3389/fnut.2022.962054

**Published:** 2022-11-16

**Authors:** José-Miguel Guzmán-García, Manuel Romero-Saldaña, Guillermo Molina-Recio, Carlos Álvarez-Fernández, Elena Raya-Cano, Rafael Molina-Luque

**Affiliations:** ^1^Department of Nursing, Pharmacology and Physiotherapy, Faculty of Medicine and Nursing, University of Cordoba, Córdoba, Spain; ^2^Lifestyles, Innovation and Health Research Associate Group, Maimonides Biomedical Research Institute of Cordoba (IMIBIC), Córdoba, Spain; ^3^Department of Occupational Health and Safety, Cordoba City Council, Huerto de San Pedro el Real, Córdoba, Spain

**Keywords:** metabolically healthy obesity, anthropometric indices, obesity, abdominal obesity, working population

## Abstract

Approximately one-third of overweight individuals, and half of those with obesity, do not have cardiometabolic disorders. For this reason, a phenotype called metabolically healthy obese (MHO) has emerged to describe this population group. The early detection of this situation could save costs associated with the development of comorbidities or pharmacological interventions. Therefore, the aim is to know the prevalence of MHO in the working population and propose variables for its detection. Cross-sectional descriptive study of 635 workers of the Cordoba City Council was carried out based on the results of the 2016 health surveillance. The outcome variables were the MHO, established based on the criteria of the IDF, NCEP—ATP III, and Aguilar—Salinas. In addition, the degree of agreement between the different MHO criteria was studied using Cohen's kappa (k), and the predictive capacity of the anthropometric variables was assessed with Receiver Operator Curves. The prevalence of MHO ranged from 6.6 to 9%. The highest agreement was reached between the IDF and NCEP-ATP III definitions (*k* = 0.811; 95% CI 0.724–0.898; *p* < 0.001). The waist-to-height ratio (WHtR) showed the highest discriminant capacity for MHO, with its best cut-off point at 0.55 for all criteria used. Sensitivity ranged from 84 to 93%. The prevalence of MHO in the working population differed according to the criteria used for diagnosis. The anthropometric variable with the highest discriminant capacity for MHO was WHtR, presenting the same cut-off point in the three criteria analyzed. Therefore, WHtR is the variable that best detects the presence of MHO.

## Introduction

Obesity is defined as an excessive accumulation of adipose tissue in the body of multifactorial origin (1), which increases the risk of suffering from cardiometabolic disorders and developing chronic non-communicable diseases (NCDs) (2). The prevalence of both conditions has tripled in the last four decades, reaching 39% for overweight and 13% for obesity in 2016 (3). Furthermore, it is estimated that the proportion of people with obesity will reach 20% by 2030 (4).

However, approximately half of the overweight population and one-third of obese subjects do not have an altered metabolic profile, i.e., the presence of hyperglycaemia, dyslipidaemia, hypertension or other cardiovascular problems (4). In this context, the phenotype defined as metabolically healthy obesity (MHO) arises to differentiate it from metabolically unhealthy obese (MNHO) (4, 5). The MHO phenotype results from several underlying mechanisms and the interaction between genetics, environment and other factors dependent on the individual's behavior, such as type of diet, level of physical activity, and presence of toxic habits, among others (6). Some factors do not depend on the individual and can vary according to the characteristics of the population studied, such as gender, age and ethnicity (7, 8).

Subjects with MHO are characterized by a body mass index above 30; a fat percentage in the obese range, with a non-visceral distribution; a metabolic profile with high insulin sensitivity, a low incidence of hypertension; a favorable lipid profile and a low level of systemic inflammatory responses (9). These characteristics favor them at half the risk of developing NCDs compared to metabolically non-healthy obesity MNHO subjects (7). Despite the growing interest in MHO, there are no harmonized criteria to define it, so the implications of this phenomenon and even its existence are still a matter of debate (8, 10). Although the lack of homogeneous criteria means that the prevalence of MHO varies according to how the diagnosis is made, the proportion of MHO is high (11). In Europe, men with MHO range from 13.5% in Germany to 19% in Italy. For women, the prevalence rate ranges from 21.1% in Italy to 28.4% in the United Kingdom (4).

On the other hand, although individuals with MHO have a healthy cardiometabolic profile, several studies have shown that, over the years, they evolve toward a metabolic risk similar to the rest of the MNHO population (11, 12). This situation is mainly due to an onset of insulin resistance linked to a change in how body adipose accumulates and is distributed, leading to ectopic visceral fat deposition (13). Furthermore, the metabolically healthy population with normal weight has a significantly lower cardiometabolic disease risk than subjects with MHO (1). This evidence establishes that no degree of obesity is healthy and certifies the need for intervention to reduce the proportion of adipose tissue. Leaving obesity aside, subjects with metabolic disturbances always have an unfavorable prognosis and higher mortality, representing the most severe phenotypic subtype (2).

Therefore, evidence shows that the MHO condition is not static (14, 15), and it is necessary to anticipate the transition to the MNHO state. In order to identify MHO early and thus avoid the transition mentioned above, the observation of anthropometric indicators can be used (11). Furthermore, early detection through these indicators could facilitate intervention through health education (healthy diet, increased physical activity or smoking cessation) that helps the population to acquire beneficial habits. Finally, these measures could avoid transitioning from MHO to MNHO (16).

Given the above, this study aims to determine the prevalence of MHO according to different diagnostic criteria, determine the clinical concordance between these criteria and propose anthropometric variables that help early detection.

## Materials and methods

### Design population and sample

A descriptive cross-sectional study was carried out on the working population of Cordoba City Council. The analysis was based on the results of the health surveillance programme in 2016, in which occupational health examinations were carried out on workers. The mean population was 1,782 workers.

Therefore, the minimum sample size calculation was 507 workers, for an expected prevalence of MHO of 8% (4), 95% confidence and a precision of 2%. The researchers randomly selected the sample and stratified it by age and sex. Initially, a total of 667 workers were selected.

All workers with a complete occupational health examination (socio-demographic variables, lifestyle, anthropometry, biochemistry and blood cell count) were included in the study. Workers with leukocytosis (leukocytes >12,000 cells/mm^3^) or hospital admission due to illness 6 months prior to the occupational health examination were excluded. The final sample consisted of 635 workers.

### Variables and measurement

The outcome variable of the study was MHO established through different diagnostic criteria: Aguilar-Salinas (17), IDF (18), and NCEP-ATP III (19) (Table 1) being mandatory in all three that the worker presents obesity (BMI ≥ 30 kg/m^2^).

**Table 1:** Diagnostic criteria for metabolic healthy obesity.

**Variable**	**Aguilar-salinas**	**NCP-ATP III**	**IDF**
Blood pressure (mmHg)	SBP < 140 and DBP < 90	SBP < 130 and/or DBP < 85	SBP < 130 and/or DBP < 85
Triglycerides (mg/dl)	≤150	<150	<150
HDL-C (mg/dl)	≥40	>40 (M) >50 (F)	>40 (M) >50 (F)
Basal glucose (mg/dl)	<126	<100	<100
WC (cm)	-	<102 (M) <88 (F)	<94 (M) or BMI <30 kg/m^2^ <80 (F) or BMI <30 kg/m^2^
Number of criteria	All the above	≥3 of the above	≥3 of the above

WC, waist circumference; SBP, systolic blood pressure; DBP, diastolic blood pressure; M, male; F, female; BMI, body mass index.

On the other hand, the independent variables collected were:

- **Sociodemographics:** Age (years), sex (male and female), job position (administrative, trades, security and others).
- **Lifestyle:** Physical activity was classified according to the short version of the International Physical Activity Questionnaire (IPAQ) (20). This tool establishes three classification groups: light, moderate and high. The light category was retained in the study, and moderate and high were unified. Tobacco habit (non-smokers, ex-smokers and smokers), and alcohol consumption according to the number of standard drinking units (SBU) per week (light-abstainer: 0–17 in men, 0–11 in women; and moderate-high: 17–28 in men, 11–17 in women) (21).
- **Anthropometric variables:** Weight (kg), height (cm), BMI (kg/m^2^), waist circumference (WC, cm), waist-to-hip ratio (WHC), waist-to-height ratio (WHtR), and body fat percentage (BF%) using the ECORE-BF equation (22). BMI categorization followed the cut-off points proposed by the World Health Organization (23).
- **Clinical variables:** SBP (mmHg) and DBP (mmHg). Blood pressure ≥ 140/90 mmHg (24) was considered high blood pressure (HBP). Diagnosis of Type 2 Diabetes Mellitus (T2DM). Metabolic Syndrome (MetS) was diagnosed through harmonized definition (25), that required to have at least three of the next criteria: WC ≥ 94 cm in men or ≥80 cm in women; Triglycerides (TG) ≥150 mg/dl or pharmacological treatment for TG; HDL-cholesterol < 40 mg/dl in men or < 50 mg/dl in women or pharmacological treatment for HDL-cholesterol; elevated blood pressure (SBP > 130 mmHg and/or DBP > 85 mmHg) or antihypertensive drug treatment; fasting plasma glucose (FPG) ≥100 mg/dl.
- **Analytical variables:** FPG (mg/dl), HDL-cholesterol (mg/dl) and TG (mg/dl).

Anthropometric measurements were collected following the recommendations of the standardized anthropometry manual (26). The researchers measured weight and height with an Atlántida S11 stadiometer and scale, with an accuracy of 0.1 kg and 0.1 cm, respectively. Finally, WC was determined at the midpoint between the last rib and the iliac crest at the end of normal expiration, and HC at the most prominent point of the buttock area. Both measurements were taken with the worker standing, feet together, using a flexible tape. On the other hand, blood pressure was taken following the recommendations established in the *Manual de Hipertensión Arterial de la Sociedad Española de Medicina de Familia* (27), using a calibrated digital sphygmomanometer (OMRON M3, OMRON Healthcare Europe, Spain). All measurements were taken by specialized personnel to minimize the coefficient of variation, each measurement was repeated three times, and the mean was calculated.

For the biochemical variables, blood samples were collected at the workplace after 12 h of fasting, from 10 ml of blood taken by venous puncture in the antecubital fossa of the arm, using a disposable vacuum tube. Once extracted, they were centrifuged between 30 and 60 min after collection and refrigerated between 2–8°C until transport and reception by the reference laboratory. They were analyzed following standardized, automated procedures in clinical biochemistry (ILAB-60 autoanalyser). The clinical analysis laboratory is accredited with the regulations' external and internal quality certifications.

### Ethical and legal aspects

All workers were informed, verbally and in writing, about the objectives of the health study to which they were subjected, and informed consent was obtained following the provisions of Law 41/2002, of 14 November, the fundamental law regulating patient autonomy and the rights and obligations regarding clinical information and documentation. The study protocol complied with the Declaration of Helsinki for human subjects' medical research and was approved by the Cordoba Bioethics Committee (4,427/Minute number 295).

### Statistical analysis

Quantitative variables have been presented with mean and standard deviation, and qualitative variables with frequencies and percentages.

To test the goodness of fit to a normal distribution of quantitative variables, the Kolmogorov-Smirnov test with the Lilliefors correction was used. The student's *t*-test was used, with prior verification of homoscedasticity of variances using Levene's test, was computed to compare two arithmetic means. The Chi-squared test and Fisher's exact test were used when necessary to compare proportions, using for the comparison of independent proportions the Z-test with bilateral hypothesis testing and a 95% confidence interval. Finally, researchers computed Cohen's Kappa index and the 95% confidence interval to determine clinical concordance between the different MHO criteria.

Receiver operator curves (ROC) were performed, and the area under the curve (AUC) was calculated to determine the discriminant ability of the independent variables for the MHO. Each predictor variable's cut-off points were determined according to the best Youden index (J). Sensitivity, specificity, predictive values, likelihood ratios and diagnostic validity were used to study diagnostic tests.

For all statistical analyses, a probability of alpha error of <5% (*p* < 0.05) was accepted and the confidence interval was calculated at 95%. SPSS (version 22.0), y EPIDAT (version 3.1 and 4.2) software were used for statistical analysis.

## Results

### Characteristics of the sample

Of the 635 workers, 67.9% were male, ranging in age from 22 to 66 years for the total population.

All anthropometric variables analyzed showed significant differences according to sex (Table 2). Regarding the prevalence of the pathologies included in the study, HBP was present in 32.4% (95% CI% 28.8–36.1%) of the workers, T2DM in 5.7% (95% CI 3.9–7.5%), and MetS in 14.3% (95% CI 11.6–17.1%). All the clinical entities discussed were more prevalent in men than in women (*p* < 0.01).

**Table 2:** Description of the sample according to study variables.

**Variable**	**Total** **(*n* = 635)**	**Men** **(*n* = 431)**	**Women** **(*n* = 204)**	***P***
Age (years)	45.1 (8.8)	46.1 (8.9)	42.8 (8)	<0.001
Physical activity		
Low	197 (31)	123 (28.5)	74 (36.3)	0.049
Moderate-High	438 (69)	308 (71.5)	130 (63.7)	0.049
Smoking habit	
Smoker	182 (28.7)	117 (27.1)	65 (31.9)	0.220
Non-smoker	327 (51.5)	213 (49.4)	114 (55.9)	0.128
Fomer smoker	126 (19.8)	101 (23.4)	25 (12.3)	<0.01
Alcohol consumption		
Abstemious-light	483 (76.1)	290 (67.3)	193 (94.6)	<0.001
Moderate-high	152 (23.9)	141 (32.7)	11 (5.4)	<0.001
Workplace		
Administrative/Office	267 (42)	113 (26.2)	154 (75.5)	<0.001
Security	122 (18.9)	113 (26.2)	9 (4.4)	<0.001
Crafts	102 (16.1)	89 (20.6)	13 (6.4)	<0.001
Other	144 (22.7)	116 (27)	28 (13.7)	<0.001
BF (%)	29.1 (6.6)	27.4 (5.4)	32.8 (7.2)	<0.001
BMI (kg/m^2^)	26.5 (4.2)	27.4 (3.9)	24.8 (4.2)	<0.001
Obesity	111 (17.5)	91 (21.1)	20 (9.8)	<0.001
WC (cm)	87.8 (12.2)	92.8 (10.1)	77.1 (9.1)	<0.001
WHC	0.88 (0.09)	0.92 (0.06)	0.79 (0.06)	<0.001
WHtR	0.52 (0.07)	0.54 (0.06)	0.48 (0.06)	<0.001
Type II diabetes mellitus	36 (5.7)	32 (7.4)	4 (2)	<0.01
Arterial hypertension	206 (32.4)	173 (40.1)	33 (16.2)	<0.001
Fasting glucose (mg/dL)	96.7 (19.4)	99.6 (21.5)	90.5 (11.8)	<0.001
HDL cholesterol (mg/dL)	56.6 (14.7)	53.3 (12.8)	63.8 (15.8)	<0.001
Tryglicerides (mg/dL)	118 (77.2)	131.2 (85.3)	90.3 (45.2)	<0.001
MetS	91 (14.4%)	75 (17.5)	16 (7.9)	<0.01
MHO	
Aguilar—Salinas	42 (6.6)	34 (7.9)	8 (3.9)	0.060
NCEP—ATP III	57 (9)	45 (10.4)	12 (5.9)	0.061
IDF	40 (6.3)	29 (6.7)	11 (5.4)	0.517

Quantitative variables: Median (Standard deviation).

Qualitative variables: Frequency (percentage).

BMI, Body Mass Index; BF, Body Fat; WC, Waist Circumference; WHC, Waist-to-Hip ratio; WHtR, Waist-to-height ratio; MetS, Metabolic Syndrome.

Concerning MHO, the prevalence varied according to the criteria used. However, no significant differences were observed between the three criteria. *For the total sample studied, the prevalence of MHO was 6.6% according to the Aguilar-Salinas criteria, 9% for NCEP-ATP III, and 6.3% for IDF*. Among workers with obesity, the prevalence was 37.8% for the Aguilar—Salinas criteria, 51.4% for NCEP—ATP III, and 36% for IDF.

Table 3 shows how the MHO is distributed among the lifestyle variables studied, with no significant differences.

**Table 3:** Distribution of the MHO according to the variables identified in the study.

**Variable**	**Aguilar-salinas** ***n* (%)**	**NCEP-ATP III** ***n* (%)**	**IDF** ***n* (%)**	p${}_{1}^{*}$	p${}_{2}^{*}$	p${}_{3}^{*}$
Physical activity	
Low	18 (42.9)	22 (38.6)	19 (47.5)	0.669	0.382	0.673
Moderate-high	24 (57.1)	35 (61.4)	21 (52.5)	0.669	0.382	0.673
Smoking habit				
Smoker	12 (28.6)	12 (21.1)	8 (20)	0.388	0.900	0.366
Non-smoker	16 (38.1)	25 (43.9)	19 (47.5)	0.565	0.723	0.389
Fomer smoker	14 (33.3)	20 (35.1)	13 (32.5)	0.856	0.791	0.936
Alcohol consumption	
Abstemious-light	26 (61.9)	38 (66.7)	30 (75)	0.624	0.378	0.203
Moderate-high	16 (38.1)	19 (33.3)	10 (25)	0.624	0.378	0.203
Workplace						
Administrative/	10 (23.8)	16 (28.1)	15 (37.5)	0.634	0.327	0.178
Office						
Security	9 (21.4)	14 (24.6)	7 (17.5)	0.715	0.406	0.654
Crafts	10 (23.8)	13 (22.8)	7 (17.5)	0.907	0.525	0.481
Other	13 (31)	14 (24.6)	11 (27.5)	0.480	0.745	0.731

Qualitative variables: frequency (percentage of workers with MHO for each criterion in each category of the study variables).

p_1_*, comparative p-value for Aguilar-Salinas / NCEP-ATPIII; P2*, comparative p-value for NCEP-ATP III / IDF; p_3_*, comparative p-value for Aquilar Salinas / IDF.

### Clinical concordance between MHO diagnostic criteria

The highest degree of agreement was observed between the criteria set out by the IDF and NCEP-ATP III (*k* = 0.811; 95% CI 0.724−0.898; *p* < 0.001), followed by that shown by the NCEP ATP III and Aguilar-Salinas definitions (*k* = 0.617; 95% CI 0.500−0.730; *p* < 0.001), and Aguilar-Salinas and IDF (*k* = 0.557 95% CI 0.424−0.690; *p* < 0.001).

Finally, the comparison of the three criteria reached a kappa value of 0.666 (95% CI 0.579−0.753; *p* < 0.001).

### Discriminating capability and diagnostic accuracy

**Figure 1:**
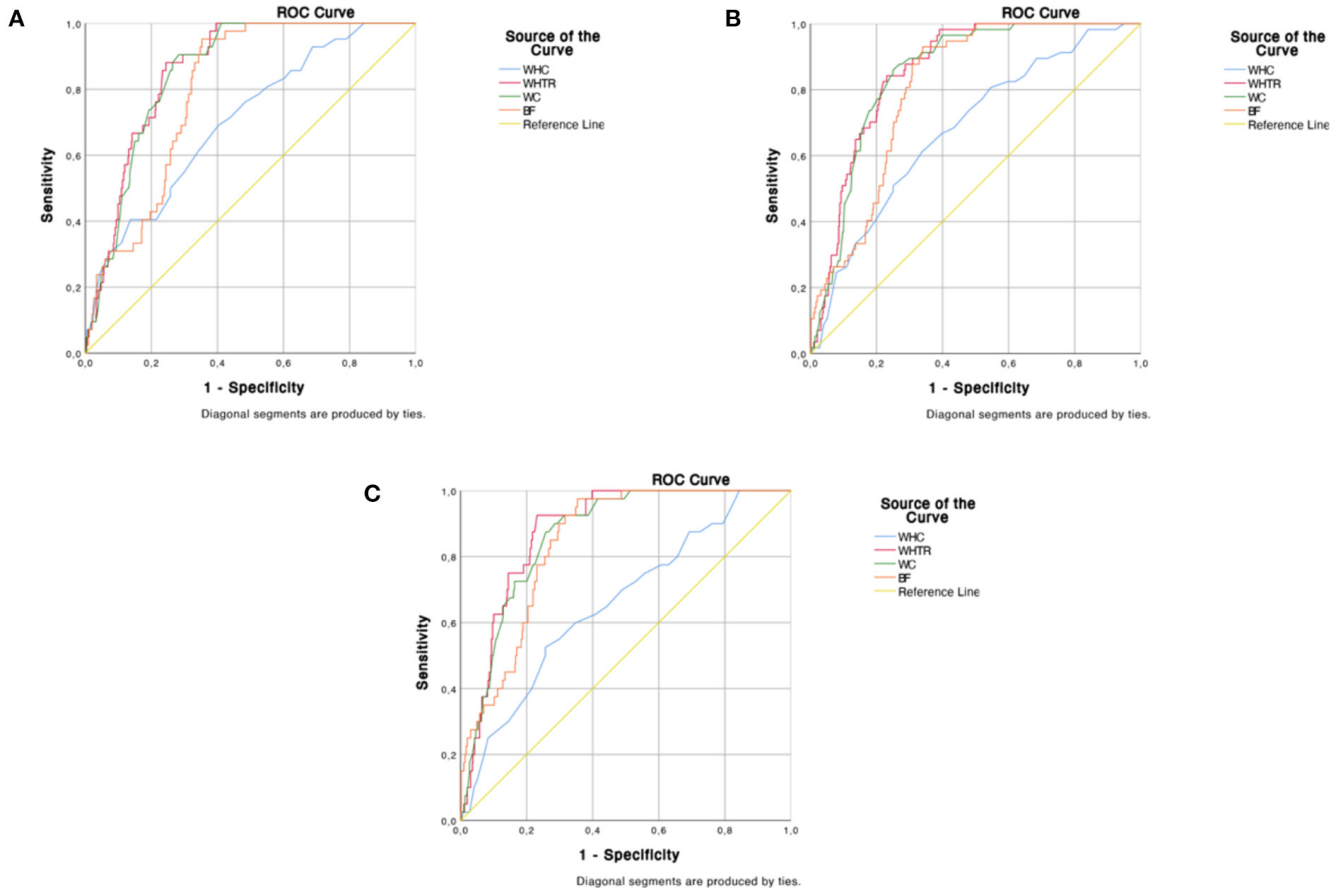
Discriminant capability of the anthropometric indicators for Metabolic Healthy Obesity according to different criteria. **(A)** Aguilar - Salinas, **(B)** NCEP-ATP III; **(C)** IDF.

WHtR was the indicator with the highest discriminant capability for MHO, using a cut-off point of 0.55. Depending on the criteria used, it showed a sensitivity between 84 and 93 % and a specificity between 76 and 77 % (Table 4, Figure 1).

**Table 4:** Discriminant capacity and diagnostic accuracy of anthropometric indicators for MHO.

**IDF critera**										
**Variable**	**AUC**	**95%CI**	***p-value***	**COV**	**Se**	**Sp**	**J**	**PPV**	**PNV**	**VI**
WHR	0.66	0.57–0.74	<0.01	0.93	0.53	0.74	0.27	0,122	0.958	0.730
WHtR	0.88	0.84–0.92	<0.001	0.55	0.93	0.77	0.69	0.211	0.994	0.778
WC (cm)	0.87	0.83–0.91	<0.001	94.90	0.88	0.74	0.62	0.186	0.989	0.751
BF (%)	0.84	0.80–0.89	<0.001	30.12	0.98	0.65	0.62	0.156	0.997	0.666
**NCEP-ATP III criteria**										
**Variable**	**AUC**	**95%CI**	***p-value***	**COV**	**Se**	**Sp**	**J**	**PPV**	**NPV**	**VI**
WHR	0.68	0.61–0.75	<0.001	0.92	0.61	0.66	0.28	0.154	0.945	0.657
WHtR	0.86	0.82–0.89	<0.001	0.55	0.84	0.77	0.61	0.264	0.980	0.775
WC (cm)	0.85	0.81–0.89	<0.001	94.25	0.86	0.75	0.61	0.254	0.982	0.760
BF (%)	0.81	0.77–0.85	<0.001	30.12	0.93	0.66	0.59	0.213	0.990	0.683
**Aguilar-salinas criteria**										
WHR	0.70	0.63–0.78	<0.001	0.91	0.69	0.60	0.29	0.109	0.964	0.604
WHtR	0.86	0.82–0.90	<0.001	0.55	0.88	0.76	0.64	0.203	0.989	0.763
WC (cm)	0.85	0.82–0.89	<0.001	93.85	0.91	0.72	0.62	0.185	0.991	0.730
BF (%)	0.80	0.75–0.84	<0.001	30.12	0.95	0.65	0.60	0.161	0.995	0.667

AUC, area under the curve; 95%CI, 95% confidence interval; COV, cut-off value; Se, sensitivity; Sp, specificity; J, Youden index; PPV, positive predictive value; NPV, negative predictive value; CC%, validity index; WHR, waist-to-hip ratio; WHtR, waist-to-height ratio; WC, waist circumference; BF, body fat percentage.

The results shown in Table 5 show that the diagnostic capability and the best cut-off point of the WHtR remain constant for the three MHO criteria used and for the diagnosis of MetS according to the harmonized definition. The most considerable difference was observed in the PPV, growing with the increasing prevalence of the clinical entity studied.

**Table 5:** Comparison of goodness of fit and diagnostic accuracy of waist-to-height ratio and body fat percentage for Metabolic Syndrome and Metabolically Healthy Obesity.

**Clinical entity**	**AUC (95%CI)**	**Cut-off point**	**Se**	**Sp**	**J**	**PPV**	**NPV**	**VI**	**PLR**	**NLR**
**Metabolic Syndrome**										
WHtR	0.89 (0.86−0.93)	0.55	0.857	0.82	0.68	0.443	0.971	0.824	4.7	0.17
BF%	0.81 (0.77−0.85)	30.37	0.835	0.71	0.54	0.323	0.962	0.724	2.8	0.23
**Aguilar-salinas**										
WHtR	0.86 (0.82–0.90)	0.55	0.88	0.76	0.64	0.203	0.989	0.763	3.6	0.16
BF%	0.80 (0.75–0.84)	30.12	0.95	0.65	0.6	0.161	0.995	0.667	2.7	0.07
**NCEP-ATPIII**										
WHtR	0.86 (0.82–0.89)	0.55	0.84	0.77	0.61	0.264	0.98	0.775	3.6	0.21
BF%	0.81 (0.77–0.85)	30.12	0.93	0.66	0.59	0.213	0.99	0.683	2.7	0.11
**IDF**										
WHtR	0.88 (0.84–0.92)	0.55	0.93	0.77	0.69	0.211	0.994	0.778	4	0.1
BF%	0.84 (0.80–0.89)	30.12	0.98	0.65	0.62	0.156	0.997	0.666	2.7	0.04

AUC, Area under the curve; Se, Sensitivity; Sp, Specificity; J, Youden ratio; PPV, Positive predictive value; NPV, Negative predictive value; VI, Validity index; PLR, Positive likelihood ratio; NLR, Negative likelihood ratio; NCEP-ATP III, The National Cholesterol Education Program, Adult Treatment Panel III; IDF, International Diabetes Federation; WHtR, Waist-to-height ratio; BF%, Percentage of fat weight.

## Discussion

The study aimed to determine the prevalence of MHO through different diagnostic criteria, evaluating the clinical concordance between them. In addition, the aim was to determine the discriminating capability of various anthropometric variables concerning this clinical entity.

Our study found a prevalence of obesity of 17.5%, being higher in men than in women (21.1% vs. 9.8%, *p* < 0.001). An analysis of 10 cohort studies in the general European population, which did not include the Spanish population (4), found a similar prevalence of obesity (17.2%), but with a higher proportion in women (18.3%) than in men (15.8%). On the other hand, a study in a Spanish working population found a higher prevalence of overweight/obesity in men than in women, a result in line with our findings (28). Similarly, Goday-Arnó et al., in their research in Spain, found an obesity prevalence of 14.9%, with the same trend in the difference between men (17.6%) and women (8.2%) (29).

Concerning MHO, the study population showed a prevalence ranging from 6.6% to 9%, depending on the diagnostic criteria. Regardless of the criteria used, these figures are lower than those found in other populations. Several studies have shown that, in the general population, the figures vary between 10% and 25% and can be as high as 47% (11, 13). In Europe, the study by Van Vliet-Ostaptchouk et al. (4) found the highest prevalence of MHO in men in the Italian cohort (19%) and women in the United Kingdom (23.1%), with the lowest prevalence found in the Finnish cohorts (4).

In the obese population worldwide, the percentage of MHO ranges from 35% (7) to 55.2%, according to the NCEP-ATP III criteria (9). Seo et al. identified a proportion of 6.8% in the Swedish population according to the Aguilar-Salinas criteria and 30.2% according to the NCEP-ATP III criteria (13). In the Korean population, following the NCEP-ATP III criteria, 24.2% of individuals with MHO were found in the obese population (30). Our research did not include the general population but exclusively the working population. A higher percentage of obese individuals with MHO was found, with 36% for the IDF criteria, 57.7% for NCEP-ATP III and 37.8% as established by Aguilar-Salinas. Recall that obesity, defined by anthropometric conditions (BMI ≥ 30), is one of the necessary conditions for diagnosing MHO in our study. However, paradoxically in the IDF criteria, obesity decreases the probability of discriminating MHO subjects, which explains why it identifies the lower number of these subjects. Meanwhile, other criteria where the anthropometric diagnosis of obesity does not have an impact, such as the NCEP-ATP III criteria, discriminate the highest number of MHO subjects.

A recent meta-analysis of cross-sectional studies concluded that subjects with MHO are more physically active, spend less time in sedentary activities and have higher cardiorespiratory fitness than MNHO (31). This finding could explain the higher prevalence of MHO individuals in our study population (exclusively at work) than the results obtained in other studies on the general population. Whether due to the requirements of their work activity or having an occupation in itself, among other variables, the working population enjoys better metabolic health.

A systematic review of different variables across studies reported that the prevalence of MHO ranged from 6% to 75%, showing that this variation depended on socio-demographic factors, including age and gender, and showed a higher prevalence among women and younger individuals (32). Similar results were found in another systematic review with meta-analysis (7) and the study by Van Vliet-Ostaptchouk et al. (4). Nevertheless, these results are the opposite of those obtained in our study, where we found a higher prevalence in men, which increases with age.

Regarding clinical concordance, a high degree of agreement was observed between the IDF and NCEP-ATP III criteria (*k* = 0.811; 95% CI 0.724–0.898; *p* < 0.001). This may be due to the similarity between the two criteria, which only differ in the cut-off point for waist circumference, being significantly lower for the IDF definition. Furthermore, a BMI ≥ 30 kg/m^2^ is a risk factor for MetS for IDF, which causes the difference in prevalence of MHO between IDF and NCEP-ATPIII. By definition, MHO requires subjects to have a BMI ≥ 30 kg/m^2^, so that, all other criteria being equal, being obese is more likely to be diagnosed with MetS following the IDF criteria than NCEP-ATP III. On the other hand, although the Aguilar-Salinas criteria have not obtained a high degree of agreement with the previous ones, it has shown that anthropometric variables discriminate well–against MHO. However, the definition used for the diagnosis does not incorporate variables of this type among its criteria.

Regarding the discriminant capability of the variables analyzed, WC and WHtR have shown the best discriminant capacity, regardless of the MHO criteria used. WHtR was the variable that achieved the highest Youden index, with a best cut-off point of 0.55. Since no other studies have addressed our research issue, it is impossible to compare the discriminant capability results for MHO.

However, a remarkable fact is that the WHtR was also the best predictor of MetS and with the same cut-off value of 0.55. Several authors have shown this point, evidencing that a WHtR of 0.55 has the best validity indicators for MetS, even when diagnostic criteria change (Harmonized, NCEP-ATP III...) (33–35). Furthermore, our results show that the probability of being diagnosed with one condition or another will depend on the PPV. This index points out that if a worker showed a WHtR >0.55, based on the PPV, he or she would be 2.2 times more likely to present MetS instead of MHO if the Aguilar—Salinas criteria are used; 1.65 times more likely, in the case of using the NCEP-ATP III criteria; and 2.1 times more likely according to the IDF.

Although BF% did not reach the discriminant capability of the WHtR, it also showed the same cut-off point in the three MHO criteria (30.12%). For this cut-off point, the Se and NPV were superior to that of the WC, offering better reliability and stability. However, these findings are not in line with those of other researchers, as no direct relationship has been found between BF% and MHO in Hispanic and Latino men (36). Likewise, it has been observed that Hispanic and Latino women with a high percentage of body fat have a lower prevalence of MHO (36).

A systematic review of prospective studies (11) to justify BMI as a predictor of MHO found that abdominal obesity was rare in subjects with a normal BMI, indicating that WC could be informative and better for stratifying the degree of overweight and obesity. Van Vliet-Ostaptchouk et al. (4) used BMI to stratify the degree of obesity and MHO in the studied population. However, they reported that other measures such as waist circumference or waist-to-hip ratio could better indicate visceral fat accumulation. In fact, in the same study, more than 95% of participants with obesity had increased waist circumference according to the NCEP-ATP III definition (19). These data are particularly striking when, in this study, it is shown that waist circumference and the waist-to-height ratio have a good discriminating capability and diagnostic accuracy. Besides, using other anthropometric variables such as waist-to-hip ratio or the percentage of body fat weight as predictors of MHO also proves helpful.

### Limitations

One of the main problems is the high heterogeneity in the methods used to define metabolic health. Furthermore, it has been challenging to compare our results, given that most of the studies analyse the phenomenon in the general population. In contrast, our findings have been carried out on the working population.

Furthermore, the number of studies focused on MHO is not high. However, this point is also a strength of the research, as it is a novel work. Therefore, further studies are needed to confirm the consistency and generalisability of the findings of this research.

## Conclusions

MHO prevalence in the working population is between 6.3 and 9%, depending on the diagnostic criteria used, and it is higher in men. The highest degree of agreement was found between the IDF and NCEP-ATP III criteria.

The variables studied that showed the greatest discriminant capabilities were WC and WHtR. Although they showed similar validity indices, WHtR was that with the highest diagnostic capacity. Therefore, regardless of the diagnostic criterion used, this index highlights if a cut-off value of 0.55 is set out for the three MHO methods and SMet.

However, despite not showing as high a discriminant capability as the previous ones, %BF proved to be a more reliable and stable indicator of body adiposity than WC, with identical cut-off values (BF ≥ 30.12%) for the three MHO methods.

## Data availability statement

The raw data supporting the conclusions of this article will be made available by the authors, without undue reservation.

## Ethics statement

The study protocol was approved by the Cordoba Bioethics Committee (4,427/Minute number 295). The patients/participants provided their written informed consent to participate in this study.

## Author contributions

J-MG-G, MR-S, CÁ-F, and RM-L made substantial contributions to conception and design, acquisition of data, analysis, and interpretation of data. J-MG-G, GM-R, and ER-C involved in drafting the manuscript or revising it critically for important intellectual content. J-MG-G, MR-S, GM-R, CÁ-F, ER-C, and RM-L given final approval of the version to be published, each author should have participated sufficiently in the work to take public responsibility for appropriate portions of the content, and agreed to be accountable for all aspects of the work in ensuring that questions related to the accuracy or integrity of any part of the work are appropriately investigated and resolved. All authors contributed to the article and approved the submitted version.

## Conflict of interest

The authors declare that the research was conducted in the absence of any commercial or financial relationships that could be construed as a potential conflict of interest.

## Publisher's note

All claims expressed in this article are solely those of the authors and do not necessarily represent those of their affiliated organizations, or those of the publisher, the editors and the reviewers. Any product that may be evaluated in this article, or claim that may be made by its manufacturer, is not guaranteed or endorsed by the publisher.
